# First Trimester Serum Copper or Zinc Levels, and Risk of Pregnancy-Induced Hypertension

**DOI:** 10.3390/nu11102479

**Published:** 2019-10-16

**Authors:** Małgorzata Lewandowska, Stefan Sajdak, Wojciech Marciniak, Jan Lubiński

**Affiliations:** 1 Division of Gynecological Surgery, Poznań University of Medical Sciences, 60-535 Poznań, Poland; ssajdak@ump.edu.pl; 2 Department of Genetics and Pathology, International Hereditary Cancer Center, Pomeranian Medical University, 71-252 Szczecin, Poland; wojciech.marciniak90@gmail.com (W.M.); lubinski@pum.edu.pl (J.L.)

**Keywords:** copper, zinc, pregnancy, hypertension, risk, BMI, smoking, microelement, trace elements

## Abstract

Early identification of women at risk of developing pregnancy-induced hypertension (PIH) is very important. The involvement of copper (Cu) and zinc (Zn) in the oxidative balance suggests the possibility of their association with this disease, in which oxidative stress plays a key role. However, it has not been established so far whether the microelement levels in early pregnancy may be risk markers of the disease, as prospective studies are limited in number. In our innovative single-center study, we identified from a prospective cohort of healthy women in the 10–14th week of a single pregnancy: women subsequently developing pregnancy-induced hypertension (*n* = 121) and matched women remaining normotensive (*n* = 363). We measured the concentrations of microelements in the serum from 10–14 week, using the inductively coupled plasma mass spectrometry (ICP-MS). The odds ratios of the disease (and 95% confidence intervals) were assessed in logistic regression. In the whole cohort, the odds ratio (OR) of PIH was 1.52 (*p* = 0.174) for women in the lowest (Q1) quartile of Cu (≤1540.58 µg/L) compared with women in the highest (Q4) quartile (>1937.46 µg/L), but adjusted odds ratio (AOR) was 2.17 (*p* = 0.019) after adjusted for pre-pregnancy body mass index (BMI) and gestational age at recruitment. The higher levels of Cu in the subgroup of BMI ≥ 25 kg/m^2^ compared to normal BMI were found (1847.64 vs. 1673.36 µg/L; *p* < 0.0001). In the subgroup of women with the normal pre-pregnancy BMI, the adjusted odds ratio of PIH was AOR = 2.95 (*p* = 0.040) for Q1 vs. Q4 quartile. Our results suggest that lower Cu levels in early pregnancy may be connected with higher risk of PIH, but BMI affected estimated odds ratios. Zinc levels had no effect on the risk.

## 1. Introduction

Copper (Cu) and zinc (Zn) are very important in human health. Both (copper and zinc) are involved, inter alia, in the oxidative balance, in division and differentiation cells, in inflammatory and immune processes and in the activation or inhibition of numerous enzymes [1,2,3,4,5]. Both are part of the Cu, Zn-DOS enzyme (superoxide dismutase), which is the enzyme of the first line of antioxidant defense [6,7,8]. Copper belongs to the transition elements and its excess may promote the intensification of oxidative stress [9]. The involvement of copper and zinc in the oxidative balance suggests the possibility of their association with pregnancy-induced hypertension, in which oxidative stress plays a key role [10,11]. Early identification of women at risk of developing pregnancy-induced hypertension is among the most important objectives of prenatal care, but there is currently no reliable biomarkers.

Pregnancy-induced hypertension (PIH) occurs on average in 5–10% of pregnant women and increases the morbidity and mortality of mothers and fetuses. This disease includes two main forms: gestational hypertension (GH) and preeclampsia (PE) [12]. Among the recognized clinical risk factors are: chronic hypertension, multiple pregnancy, primiparity, and pre-pregnancy obesity [13]. The disease is characterized by arterial pressure increase de novo after the 20th week. It has been shown that abnormal invasion of trophoblasts (in the first half of pregnancy) results in insufficient re-modelling of the spiral arteries, which leads to placental high-resistance circulation and hypoxia [12,13,14,15]. This results in intensification of the production of ROS (reactive oxygen species) and intensification of oxidative stress Antioxidant enzymes (including Cu, Zn-DOS) protect trophoblast cells against oxidative stress [10,11]. Lower Cu and Zn levels may be associated with lower antioxidant activity and higher risk of the disease. 

The meta-analyses of retrospective studies showed that women with preeclampsia had higher levels of copper [16,17] or lower levels of zinc [18,19,20], compared to normotensive women, however, the microelement status in women who have developed the disease may be a result of existing disorders. There are limited prospective studies in this topic [10,21,22,23]. In a small cohort of 151 women, Basu et al. found non-statistically lower plasma Cu levels in 12.2 ± 1.9 week in 23 women who subsequently developed preeclampsia compared to 24 controls; but they also found higher Zn concentrations [22]. In a cohort of 349 women, Ugwuja et al. found statistically lower plasma Cu levels in <25th week in 40 women developing preeclampsia compared to controls [21]. In contrast, Mistry et al. in a multicenter study, found statistically higher plasma Cu levels in 15 ± 1 week in women who developed preeclampsia (*n* = 244) compared to controls [10]. Importantly, it was shown that obesity, a risk factor of PIH, is associated with oxidative stress and inflammation and with higher concentrations of copper [9,10]. It has not been investigated whether different maternal BMI categories can affect PIH odds ratios depending on the microelement concentrations.

The problem remains open to whether copper or zinc status may be risk markers of the disease. Therefore, the aim of this study is to determine the association between serum copper or zinc concentrations (and their balance) in early healthy pregnancy and the risk of pregnancy-induced hypertension. The innovative study was conducted in two matched groups, which allowed for the influence of several confounders to be excluded. The impact of pre-pregnancy BMI and smoking factors on results were taken into account. This is the first single-center study for serum levels in these microelements, conducted for such a great number of cases. 

## 2. Materials and Methods 

### 2.1. Study Population

Two matched groups of women were enrolled in this study. Data came from a prospective cohort of pregnant women, recruited in the end of the first trimester of pregnancy at the University Hospital in Poznan, Poland in 2015–2016. The study was conducted according to the Helsinki Declaration. All the participants provided written consent (signed the Informed Consent Form and the Test Information Form before submitting a blood sample). The study was approved by the Bioethics Committee of the Medical University of Poznan, Poland, under number 769/15.

The participants included in this study were healthy white women of Polish descent from the Wielkopolska region (Central Europe), in the 10th (+0)–14th (+6) week of pregnancy, aged 18–45 years at conception, a confirmed single pregnancy with normal course without aneuploidy and with delivery of a phenotypically normal child >24 gestational weeks, using a normal diet. Chronic diseases apart from being overweight or obesity (hypertension and diabetes mellitus, kidney or liver diseases, immunological or inflammatory diseases, thromboembolism) were excluded. The use of multivitamin/microelement supplementation was not the inclusion/exclusion criteria. 

The population, wherein this study was nested, consisted of 912 women who met all inclusion/exclusion criteria after delivery and reported a data set during pregnancy and after delivery. In this population, 137 women subsequently developed pregnancy-induced hypertension (cases) and 775 women remained normotensive (controls). We excluded cases where blood samples were unavailable (*n* = 16). We examined a study group consisting of cases (*n* = 121) and a matched control group consisting of normotensive women (*n* = 363). The case group included 106 cases of gestational hypertension and 15 cases of preeclampsia.

### 2.2. Method and Data Collection

Data (obstetrical and gynecological histories, concurrent diseases, medications and supplements, socioeconomic and demographic characteristics, smoking, alcohol consumption, family medical histories) were collected using a questionnaire during the recruitment. The women themselves filled out the questionnaire in the presence of midwives. Subsequently, the participants were observed until the 12th week after parturition. We contacted the participants by telephone or e-mail. Data was verified during the observation. Pregnancy outcomes were taken from the medical records. Some information was passed on by the participants themselves, such as the weight before pregnancy and the names of supplements. All women declared no alcohol in pregnancy. The socio-economic characteristics included maternal education level categories (among others, education < 12 years). “The financial status was assessed according to a 5 Lickert’s scale, on the basis of an answer to the question: Is your financial situation (in your household) good enough to meet your needs? The responses were classified in the following way: (1) definitely NO; (2) RATHER NO; (3) hard to say; (4) RATHER YES; (5) definitely YES. In this survey we distinguished: lower financial levels (1 and 2), medium (3) and higher (4 and 5). Data concerning the place of residence included the following categories: village, small town (<50 thousand inhabitants), and big city.” Only available data were taken into account. 

The normal pre-pregnancy body mass index (BMI) was defined as 18.5–24.99 kg/m^2^. Gestational weight gain was defined as the difference between the weight measured before delivery (from medical records) and the pre-pregnancy weight. 

The blood pressure was measured in a sitting position with an oscillometric device on the arm. Participants declared normal values of blood pressure before pregnancy (<140/90 mmHg) and declared values of blood pressure before recruitment (from medical records). We recorded the blood pressure noted after leaving the postpartum ward (in the maternity ward). 

### 2.3. Definition of the Studied Outcome 

Pregnancy-induced hypertension was defined in accordance with the national guidelines (2015) consistent with new definition [24], as “arterial pressure equal to and higher than 140/90 mmHg (on two occasions, at least 4 h apart) developed de novo after the 20th week of pregnancy, receding up to 12 weeks after delivery.” The disease incudes two main forms: gestational hypertension and preeclampsia. Gestational hypertension was diagnosed if no other disturbance was found. Preeclampsia was diagnosed when any of the following appeared de novo: proteinuria (≥300 mg/day or ≥0.3 g/L; protein/creatinine ratio ≥ 0.3; 1+ in the strip test); thrombocytopenia <100 G/L; worsening of renal function; damage to the liver function; pulmonary edema; symptoms from the central nervous system; blurred vision. IUGR (intrauterine growth restriction) was not a criteria of diagnosis.

In our cohort, only proteinuria (≥0.3 g/L) occurred in all cases of preeclampsia. 

### 2.4. Serum Copper and Zinc Determination

Maternal blood samples were taken at the recruitment (in 10–14 gestational week). Venous blood of cases and controls was collected into Sarstedt Monovette system (Sarstedt, Germany) using Serum Z/7.5 mL tubes. After collection, tubes were inverted several time for better clotting. Tubes were placed at room temperature at least 30 min, but no longer than 2 h to clot. Blood samples were centrifuged at 1300 G for 12 min. Patient sera were stored at −80 °C until analysis. Before performing analysis sera were thawed, vortexed, and centrifuged at 5000 G for 5 min before copper determination.

Samples copper and zinc determination was carried out with ICP (inductively coupled plasma) mass spectrometer NexION 350D (PerkinElmer, Shelton, CT, USA). Before each analytical run, the instrument was tuned to achieve manufacturers’ criteria. Measurement were performed under DRC (dynamic reaction cell) condition with methane as a reaction gas. Spectrometer was calibrated using external calibration technique. Calibration standards were prepared from 10 µg/mL multi-element calibration standard 3 (PerkinElmer, Shelton, CT, USA) by diluting with blank reagent to the final concentration of 1, 5, 10 μg/L (for measurement of Cu) and 1, 5, 10 μg/L (for measurement of Zn); correlation coefficients for calibration curves were always greater than 0.999.

Analysis protocol assumed 100-fold dilution of serum in blank reagent. Blank reagent consisted of 10 mL of 65% Suprapur Grade nitric acid (Merck, Darmstadt, Germany), 0.20 mL of Triton X-100 (PerkinElmer, Shelton, CT, USA) filled to the mark of 1-L flask with >18 MΩ/cm^2^ deionized water (Merck Millipore, Burlington, MA, USA). Germanium isotope (Ge74) was set as internal standard. 

Accuracy and precision of measurements were tested using certified reference material (CRM), Clincheck Plasmonorm Serum Trace Elements Level 1 (Recipe, Munich, Germany). Additionally, internal quality control samples were measured during analysis. General precision was lower than 5% RSD (relative standard deviation). Final concentration include dilution factor and coefficient which was mean value of two flanking certified reference materials concentrations divided by mean concentration determined by manufacturer of CRM.

### 2.5. Statistical Analyses

The data was imported into the Statistica 13 package. The normality of data distribution were checked by the Shapiro-Wilk test. The Mann-Whitney U test was used for continuous variables comparisons (the distributions were not normally and medians were compared), and Pearson’s chi-square test was used for comparisons of categorical variables (*p*-value < 0.05 was assumed to be significant). The ratios of Cu:Zn concentrations were calculated for each woman. Copper, zinc, and the Cu:Zn ratio levels were compared between the case and control groups using the Mann-Whitney U test. 

We chose the control group (*n* = 363) by individual matching cases of pregnancy-induced hypertension (*n* = 121) in relation to the following criteria (in a 3:1 ratio): maternal age (±2 years), pre-pregnancy BMI (±10%), and women who have never smoked. The age in both groups was similar but not exactly the same (*p*-value was 0.907). BMI turned out to be mismatched (was statistically different between the groups). And we calculated the odds ratios (and 95% confidence intervals CI) of pregnancy-induced hypertension in logistic regression.

We examined the whole cohort (*n* = 484) and the subgroup of normal pre-pregnancy BMI (*n* = 265). We divided the whole cohort and the subgroup into quartiles based on the distribution of the Cu or Zn or Cu:Zn ratio levels. The odds ratios of pregnancy-induced hypertension for Cu and Zn and Cu:Zn ratio levels (from 10–14 gestational week) for each quartile with regard to the highest Q4 quartile were calculated. *p*-value was calculated using the Wald test, and value <0.05 was assumed to be significant. We calculated the odds ratios in the univariate (OR) and multivariate (AOR) logistic regression.

The match ruled out the influence of several risk factors. The odds ratios (OR) were calculated in univariate logistic regression accepting that several risk factors were not statistically different between the groups (maternal age, parity, pack-years of smokers, assisted reproductive technology, diabetes mellitus at present pregnancy and vitamins/microelements supplementation). The adjusted odds ratios (AOR) were calculated in multivariate logistic regression after adjusting for confounders (risk factors that were statistically different between the groups): pre-pregnancy BMI and gestational age at recruitment (in the subgroup, the pre-pregnancy BMI was excluded). Number of prior preeclampsia was small (3 women among 121 cases of PIH) and it was not used as confounders. Clinical risk factors for pregnancy-induced hypertension [13] and relation to the level of copper and zinc [25] have been identified based on literature data.

## 3. Results

The general characteristics of the normotensive controls and cases of pregnancy-induced hypertension are presented in Table 1. In total, 484 women participated in the analysis. We examined 121 women who subsequently developed pregnancy-induced hypertension and matched 363 women who remained normotensive. The average age of women in the case group was 35.1 years (range 19–45), and in the normotensive group was 35.1 years (range 22–45) (*p* = 0.907). The mean pre-pregnancy BMI was higher in the case group compared to the normotensive women (*p* = 0.003). We found differences in pregnancy outcomes. The mean gestational age at delivery was lower in the case group compared to the normotensive women (*p* = 0.011) and the mean birthweight was lower in the case group compared to the normotensive women (*p* = 0.0003). In the whole cohort, 106 cases of gestational hypertension (GH) and 15 cases of preeclampsia (PE) were found. The clinical characteristics of the subgroup of women with the normal pre-pregnancy BMI (Table S1) were similar, and among cases of PIH, there were 47 cases of GH and 7 cases of PE.

Microelement characteristics in groups are presented in Table S2 and Table 2. In the entire cohort (Table S2), statistically insignificantly lower mean levels of copper (*p* = 0.059), zinc (*p* = 0.689), and Cu:Zn ratio (*p* = 0.320) were found in the case group compared to the normotensive women. Pre-pregnancy BMI ≥ 25 kg/m^2^ was associated with the higher levels of copper, compared to normal BMI (1847.64 vs. 1673.36 µg/L; *p* < 0.0001). Smoking were not related with changes of microelement concentrations. In the subgroup of normal pre-pregnancy BMI (Table 2), statistically significantly lower mean levels of copper (*p* = 0.008) and lower mean levels of Cu:Zn ratio (*p* = 0.037) were found in the case group compared to the normotensive women. Serum zinc levels were not related to the disease (*p* = 0.867).

The risks of the disease for serum levels of microelements in early pregnancy are presented in Table S3 and Table 3. In the whole cohort (Table S3), no statistical associations between quartiles of copper, zinc or Cu:Zn ratio and the disease risk were found in univariate logistic regression. However, the adjusted PIH odds ratio (AOR) was 2.17 (*p* = 0.019) after adjusting for pre-pregnancy BMI and gestational age at recruitment, for the lowest (Q_1_) quartile of Cu (≤1540.58 µg/L) compared to the highest (Q_4_) quartile (>1937.46 µg/L) (Table 3). In the subgroup of normal pre-pregnancy BMI (Table 3), women in the lower Q_1_ or Q_2_ quartiles of copper had about three-fold increase in pregnancy-induced hypertension risk compared to women in the highest Q_4_ quartile. Women in the lowest (Q_1_) quartile of Cu:Zn had about three-fold increase in risk compared with women in the highest (Q_4_) quartile, and Zinc levels were not related to pregnancy-induced hypertension (Table S3). These results were obtained after matched several risk factors (see in Statistical Analyses chapter). 

## 4. Discussion

There are limited prospective studies examining whether copper or zinc levels in early pregnancy may be risk markers of pregnancy-induced hypertension. Our study in matched groups showed that women in the lower quartiles (Q_1_ or Q_2_) of copper had about three-fold increase in pregnancy-induced hypertension risk, compared to women in the highest quartile. The conclusion, however, was only valid after adjusting for pre-pregnancy BMI and gestational week at recruitment (in the whole cohort). Additional analysis in the subgroup of women with the normal BMI also showed these compounds. Additionally in the subgroup, we found that the lowest ratio of Cu:Zn can be associated with higher risk of the disease. First trimester serum zinc levels had no effect on the risk. Our results may have some important implications for the early identification of women at risk of developing pregnancy-induced hypertension. 

The levels of microelements can be affected by different factors: e.g., genetics, geographical location, and content of elements in the soil, food preparation, food and supplements accessibility, ethnic differences in body composition, seasonal variation, pregnancy age, and socio-economic factors related to lifestyle indicators as incorrect diet, obesity, or smoking [25,26,27,28,29,30,31]. According to the literature, compared to non-pregnant women, copper concentrations in pregnancy are significantly increased, returning after delivery to pre-pregnancy levels. This increase is partially explained by the synthesis of ceruloplasmin, but the mechanisms have not been fully elucidated [23,32]. Serum/plasma zinc levels are reduced from the first to the third trimester, which is mainly explained by the increase in plasma volume and transfer from the mother to the fetus [23,32]. 

In our study, we have taken into account several factors influencing the levels of micronutrients and/or the risk of pregnancy-induced hypertension [13,25,27], and several confounders were matched between groups (e.g., mother’s age, parity, assisted reproductive technology, pack-years of smokers, use of multivitamin/microelement supplementation in pregnancy, and gestational diabetes mellitus at present pregnancy). Our study covered one region of Poland, which matched the women with respect to the same level of prenatal care and diet. Number of prior preeclampsia was small (3 women among 121 cases of PIH) and it was not used as confounders. We adjusted the odds ratios of the disease (PIH or GH) for pre-pregnancy BMI and gestational age at recruitment that were statistically different between the groups. In this study, among cases of PIH (*n* = 121), there were only 15 cases of preeclampsia (PE), indicating that the analysis is focused on gestational hypertension (GH). For GH cases, the results were similar to those for all PIH cases. The clinical characteristics of the cases and controls in the subgroup (of normal BMI) were similar to those in the whole cohort (Table S1).

We found association between lower serum copper concentrations in early pregnancy and pregnancy-induced hypertension. The same results were obtained by Ugwuja et al. [21] and Basu et al. [22] in smaller groups. Opposite results were obtained by Mistry et al. in the international multicenter cohort [10]. Among the reasons for the discrepancy we included differences in population risk, size of groups, degree of matching and in the biological material and measurement methods. Our results may suggest the importance of copper as an early biomarker of the risk of pregnancy-induced hypertension and suggest that further studies are necessary to determine the importance of copper deficiency among causes of the disease.

The main mechanism of associations found may be a deficiency of copper antioxidant effects in early pregnancy. It has been shown that abnormal invasion of trophoblasts in the walls of the spiral arteries between the 6th and 18th week results in cascade processes in placenta including an increase in the production of reactive oxygen species and increased oxidative stress [12,13,14,15]. The increase in oxidative stress require an increase in the activity of antioxidant systems in the placenta, including Cu, Zn-DOS, which strive to maintain an oxidative balance and protect trophoblast cells [10,33,34]. Appropriate levels of copper and zinc (and probably proper balance) are needed to ensure correct functioning of Cu, Zn-SOD [11]. In literature, the lowest levels of other microelements, selenium and iron in early pregnancy were also associated with higher risk of pregnancy-induced hypertension [35,36].

Our results have shown a relationship between lower copper levels and pregnancy-induced hypertension after the exclusion of those who are overweight/obese. In our opinion, it is connected to the higher copper levels in women with the excessive pre-pregnancy BMI (Table S2). According to literature data, obesity is associated with an increase in the concentration of copper; this increase may be consistent with the inflammatory response found in obesity [26,28,29,30]. Inflammation is associated with higher levels of ceruloplasmin, an acute phase protein that binds copper [9,10]. This increase in copper levels in obesity and/or inflammatory response could mask the deficiency of this element [37].

We found no association between serum zinc concentrations in early pregnancy and pregnancy-induced hypertension. The same results were obtained by Mistry et al. [10] and Ugwuja et al. [21]. The diet has been shown to be the main factor influencing the status of zinc, therefore our results may be a confirmation of the lack of zinc deficiency in countries with higher incomes [38]. At the same time, in our study of the subgroup of normal pre-pregnancy BMI, associations of the lowest ratio of Cu:Zn in early pregnancy with higher risk of the disease were found. Basu et al. also found lower Cu:Zn ratios in the 12.2 ± 1.9 week in women subsequently developing preeclampsia compared to controls [22]. These results may indicate a greater deficiency of the antioxidant activity of copper in relation to zinc activity. It can suggest the need to pay attention to the copper-zinc balance in women in early pregnancy or planning pregnancy. It is generally known that zinc and copper compete during absorption [39], and GWAS studies (genome-wide association study) have shown that there is a link between Cu and Zn metabolic pathways [40]. 

Our results suggest that further studies of micronutrient status in early pregnancy are necessary to determine their importance for the proper development of pregnancy.

One strength of our study was the innovative analysis in matched groups, nested in the prospective cohort. As far as we know, this is the first single-center study which investigated the serum level of copper and zinc in assessing the risk of pregnancy-induced hypertension in groups of such a large number of cases. We have taken into account many risk factors, but there may be other confounding variables. A strength was the additional analysis in the subgroup of normal pre-pregnancy BMI, however, the study in the subgroup has its limitations.

There are a few limitations, though. The evaluation of ceruloplasmin concentrations would be an interesting complement to the results. Examining the microelement levels at several time points of pregnancy would be a very interesting complement to the analysis. The participants declared having followed a normal diet, but we did not study the composition of the diet. Participants reported fasting before blood samples were taken, but in pregnant women this may be of a limited value because of the known impairment of peristalsis in pregnancy. Use of multivitamin/microelement preparations cannot be excluded in the population of pregnant women; however, in our study frequency of use of these preparations did not differ statistically significantly between the groups. A small number of women with prior preeclampsia were found; therefore the results were not adjusted for this variable. Number of cases of preeclampsia (PE) was small.

## 5. Conclusions

In this study, lower serum copper levels in early pregnancy were connected with higher risk of pregnancy-induced hypertension, however excessively high pre-pregnancy BMI influences the odds ratios of the disease and it can be a reason for the discrepancies between studies.

Our results show that pre-pregnancy BMI ≥ 25 kg/m^2^ was associated with significantly higher serum copper concentrations in the 10–14th pregnancy week, which could mask the deficiency of the element.

In our study, serum zinc levels (in the 10–14th pregnancy week) were not related to pregnancy-induced hypertension. 

All our results can suggest the need to pay attention not only to the levels of copper, but also to a copper-zinc mutual balance in women in early pregnancy or planning pregnancy. The impact of BMI on the serum copper levels must be taken into account.

## Figures and Tables

**Table 1:** The clinical characteristics of the normotensive controls and cases of pregnancy-induced hypertension.

Characteristics	Normotensives (*n* = 363) *	Cases (*n* = 121) *	
	Mean (SD) or *n* (%)	Mean (SD) or *n* (%)	*p* **
Maternal age (years)	35.1 (4.0)	35.1 (4.2)	0.907
Maternal age (range)	(22–45)	(19–45)	
Gestational age at recruitment (weeks)	12.3 (0.8)	11.6 (0.8)	1.97 × 10^−^^16^
Pre-pregnancy BMI (kg/m²)	25.0 (4.4)	26.8 (5.4)	0.003
Primiparous	141 (38.84%)	56 (46.28%)	0.149
Prior PE	−	3	−
Prior PIH/PE ^•^	2 (0.55%)	13 (10.74%)	<0.001
ART ^•^	18 (4.96%)	11 (9.09%)	0.097
Women who have never smoked	302 (83.20%)	92 (76.03%)	0.080
Pack-years of smokers ***	19.3 (32.5)	21.2 (32.3)	0.748
Multivitamins in II-III trimester	184 (50.69%)	50 (41.32%)	0.074
Education <12 years (for available data)	28 (9.18%)	20 (19.05%)	0.007
Lower financial status (1-2-3)	46 (32.6%)	31 (49.2%)	0.002 ^#^
Village	110 (30.4%)	30 (25.0%)	0.585 ^#^
Outcomes			
Gestational age at delivery (weeks)	38.7 (1.8)	37.99 (2.6)	0.011
Newborn birthweight (g)	3385.3 (546.8)	3113.1 (785.4)	0.0003
Systolic blood pressure (mmHg) ****	107.3 (11.4)	158.3 (18.2)	<0.0001
Diastolic blood pressure (mmHg) ****	66.0 (8.8)	100.2 (10.5)	<0.0001
Preeclampsia	−	15	−
Gestational hypertension	−	106	−
GDM ^•^	73 (20.11%)	23 (19.01%)	0.792

* Normotensive controls and cases of pregnancy-induced hypertension (PIH); ** The Mann-Whitney U test was used for comparisons of continuous variables and medians were compared, and the Pearson chi-square test was used for categorical variables comparisons (*p*-value < 0.05 was assumed to be significant); *** for smokers during recruitment; **** after leaving the postpartum ward; ^#^ for available data and for several categories; ^•^ PIH/PE: pregnancy-induced hypertension/ preeclampsia, ART: assisted reproductive technology, GDM: gestational diabetes mellitus.

**Table 2:** Serum copper and zinc concentrations (in 10–14 gestational week) in the normal body mass index (BMI) subgroup.

Biomarkers (µg/L) **	Normotensives *	Cases *	
	Mean (SD)	Mean (SD)	*p* ** **
Normal BMI subgroup, n ***	211	54	
Copper	1693.39 (275.70)	1595.01 (255.24)	0.008
Zinc	614.60 (93.75)	615.40 (83.60)	0.867
Cu:Zn ratio	2.81 (0.60)	2.65 (0.60)	0.037

* Cases of pregnancy-induced hypertension (PIH) and normotensive controls; ** Microelement concentrations were measured in serum from 10–14 gestational week (µg/L); *** normal pre-pregnancy BMI: body mass index 18.50–24.99 kg/m^2^; ** ** *p*-value obtained using the Mann-Whitney U test and *p* < 0.05 was assumed to be significant.

**Table 3:** The odds ratios of PIH/GH for Cu levels.

Quartile	Cu (µg/L) ^!^	Cases ^•^	Controls ^•^	Odds Ratios of PIH/GH	
				OR * (95% CI:); *p* ***	AOR ** (95% CI:); *p* ***
Whole cohort (PIH risk)					
Q_1_	883.61–1540.58	33	88	1.52 (0.83–2.76); 0.174	2.17 (1.14–4.16); 0.019
Q_2_	1540.58–1733.78	37	84	1.78 (0.99–3.22); 0.056	2.39 (1.28–4.49); 0.007
Q_3_	1733.78–1937.46	27	94	1.16 (0.63–2.16); 0.637	2.35 (0.71–2.57); 0.360
Q_4_	1937.46–3956.76	24	97	1	1
Whole cohort (GH risk)					
Q_1_	883.61–1541.95	30	76	1.60 (0.84–3.02); 0.150	2.36 (1.19–4.68); 0.015
Q_2_	1541.95–1735.86	32	74	1.75 (0.93–3.30); 0.083	2.35 (1.20–4.60); 0.012
Q_3_	1735.86–1937.46	23	83	1.12 (0.58–2.18); 0.735	1.27 (0.64–2.52); 0.503
Q_4_	1937.46–3956.76	21	85	1	1
Subgroup ^#^					
Q_1_	969.60–1486.63	17	49	2.97 (1.14–7.75); 0.026	2.95 (1.05–8.27); 0.040
Q_2_	1486.63–1667.50	18	48	3.21 (1.24–8.33); 0.016	4.05 (1.44–11.43); 0.008
Q_3_	1667.50–1838.35	12	54	1.91 (0.70–5.19); 0.208	2.18 (0.75–6.39); 0.154
Q_4_	1838.35–2500.75	7	60	1	1

^#^ Normal pre-pregnancy; BMI: body mass index 18.50–24.99 kg/m^2^; ^!^ border values are included in the lower quartile; ^•^ Cases of pregnancy-induced hypertension and normotensive controls; * OR: crude odds ratio calculated in univariate logistic regression (several risk factors were matched between groups); ** AOR: adjusted odds ratio calculated in multivariate logistic regression after additional correction by pre-pregnancy BMI and gestational age at recruitment (in the whole cohort) and by gestational age at recruitment in the subgroup; *** *p* < 0.05 was assumed to be significant; CI: confidence intervals.

## Supplementary Materials

The following are available online at https://www.mdpi.com/2072-6643/11/10/2479/s1, Table S1: The clinical characteristics of the controls and cases in the subgroup of women with the normal BMI. Table S2: Complete characteristics of copper, zinc, and Cu:Zn ratio levels (in serum from 10–14 gestational week) in the whole cohort and subgroups. Table S3: The odds ratios of pregnancy-induced hypertension for copper, zinc, and Cu:Zn ratio levels in the whole cohort and subgroups, in univariate and multivariate logistic regression.
