# Supplementary material for: First Trimester Serum Copper or Zinc Levels, and Risk of Pregnancy-Induced Hypertension
Source: Nutrients. 2019 Oct 16;11(10):2479. doi: 10.3390/nu11102479 (PMC6835641; doi:10.3390/nu11102479)
Supplement: Supplementary file 1 [file nutrients-11-02479-s001.zip › Table S1.docx]

**Table S1.** The clinical characteristics of the controls and cases in the subgroup of women with the normal BMI

|  | **Normotensives (*n* = 211)** | **Cases (*n* = 54)** |  |
| --- | --- | --- | --- |
| **Characteristics** | **Mean (SD) or *n* (%)** | **Mean (SD) or *n* (%)** | ***P* *** |
| Subgroup |  |  |  |
| Maternal age (years) | 35.0 (4.7) | 34.8 (4.7) | NS |
| Maternal age (range) | (22 – 45) | (19 – 45) |  |
| Gestational age at recruitment (weeks) | 12.3 (0.8) | 11.5 (0.8) | < 0.0001 |
| Pre-pregnancy BMI (kg/m²) | 22.2 (1.4) | 22.2 (1.3) | NS |
| Pre-pregnancy BMI (range) | (18.6 – 24.98) | (18.6 – 24.97) |  |
| Primiparous | 88 (41.7%) | 26 (48.1%) | NS |
| Prior PE | − | − | − |
| Prior GH | 2 (1%) | 8 (14.8%) | <0.0001 |
| ART • | 16 (7.6%) | 4 (7.4%) | NS |
| Women who have never smoked | 184 (87.2%) | 45 (83.3%) | NS |
| Pack-years of smokers ** | 5.2 (10.1) | 5.3 (10.2) | NS |
| Multivitamins in II-III trimester | 104 (49.3%) | 22 (40.7%) | NS |
| Outcomes |  |  |  |
| Gestational age at delivery (weeks) | 38.6 (1.7) | 38.0 (2.6) | <0.05 |
| Newborn birthweight (g) | 3326.5 (526.0) | 2988.2 (721.4) | <0.0001 |
| GH | - | 47 |  |
| PE | - | 7 |  |
| Gestational diabetes mellitus | 36 (17.%) | 7 (13.0%) | NS |

* The Mann-Whitney U test was used for comparisons of continuous variables and medians were compared, and the Pearson chi-square test was used for categorical variables comparisons (p-value < 0.05 was assumed to be significant); **for smokers during recruitment; • ART: assisted reproductive technology; GH: gestational hypertension; PE: preeclampsia, †results for all categories of the variable; NS: not statistical (p >0.05).
