# Supplementary material for: First Trimester Serum Copper or Zinc Levels, and Risk of Pregnancy-Induced Hypertension
Source: Nutrients. 2019 Oct 16;11(10):2479. doi: 10.3390/nu11102479 (PMC6835641; doi:10.3390/nu11102479)
Supplement: Supplementary file 1 [file nutrients-11-02479-s001.zip › Table S2.docx]

**Table S2.** Complete characteristics of copper, zinc and Cu:Zn ratio levels (in serum from 10-14 gestational week) in the whole cohort and subgroups.

|  |  | **Characteristics of Copper, Zinc and Cu:Zn Ratio Levels *** | | | |
| --- | --- | --- | --- | --- | --- |
| **Groups **** | ***n*** | **Mean** | **Range** | **Median** | ***p* ***** |
| **Cu (µg/L)** |  |  |  |  |  |
| **Whole cohort** |  |  |  |  |  |
| Cases | 121 | 1698.33 | 883.61–2396.43 | 1671.44 | 0.059 |
| Controls | 363 | 1767.53 | 965.46–3956.76 | 1746.67 |  |
| **Pre-pregnancy BMI categories** |  |  |  |  |  |
| BMI ≥ 25 kg/m² | 211 | 1847.64 | 883.61–3956.76 | 1814.04 | < 0.0001 |
| BMI 18.5-24.99 kg/m² | 265 | 1673.36 | 969.60–2500.75 | 1671.15 |  |
| **Smoking categories** |  |  |  |  |  |
| Smokers at recruitment | 32 | 1716.39 | 1204.22–2500.75 | 1634.63 | 0.419 |
| Women who have never smoked | 394 | 1750.44 | 883.61–3956.76 | 1733.85 |  |
| **Subgroup of BMI ≥ 25 kg/m²** |  |  |  |  |  |
| Cases | 66 | 1777.14 | 883.61–2374.11 | 1787.36 | 0.162 |
| Controls | 145 | 1879.73 | 965.46–3956.76 | 1826.63 |  |
| **Subgroup of normal BMI #** |  |  |  |  |  |
| Cases | 54 | 1595.01 | 1042.92–2396.43 | 1562.08 | 0.008 |
| Controls | 211 | 1693.39 | 969.60–2500.75 | 1696.98 |  |
| **Zn levels * (µg/L)** |  |  |  |  |  |
| **Whole cohort** |  |  |  |  |  |
| Cases | 121 | 610.19 | 394.04–917.84 | 607.66 | 0.689 |
| Controls | 363 | 628.03 | 406.11–3238.90 | 607.49 |  |
| **Pre-pregnancy BMI categories** |  |  |  |  |  |
| BMI ≥ 25 kg/m² | 211 | 633.09 | 394.04–3238.90 | 608.87 | 0.969 |
| BMI 18.5-24.99 kg/m² | 265 | 614.76 | 411.94–1069.54 | 607.23 |  |
| **Smoking categories** |  |  |  |  |  |
| Smokers at recruitment | 32 | 630.95 | 478.22–917.84 | 630.73 | 0.275 |
| Women who have never smoked | 394 | 626.02 | 406.11–3238.90 | 607.15 |  |
| **Subgroup of BMI ≥ 25 kg/m²** |  |  |  |  |  |
| Cases | 66 | 607.01 | 394.04–812.82 | 607.20 | 0.552 |
| Controls | 145 | 644.96 | 406.11–3238.90 | 609.61 |  |
| **Subgroup of normal BMI #** |  |  |  |  |  |
| Cases | 54 | 615.40 | 411.94–917.84 | 616.02 | 0.867 |
| Controls | 211 | 614.60 | 424.86–1069.54 | 607.07 |  |
| **Cu:Zn ratio** |  |  |  |  |  |
| **Whole cohort** |  |  |  |  |  |
| Cases | 121 | 2.858 | 1.290–5.423 | 2.810 | 0.320 |
| Controls | 363 | 2.908 | 0.535–5.989 | 2.885 |  |
| **Subgroup of normal BMI #** |  |  |  |  |  |
| Cases | 54 | 2.647 | 1.588–4.559 | 2.644 | 0.037 |
| Controls | 211 | 2.814 | 1.181–4.645 | 2.784 |  |

# normal body mass index: 18.5–24.99 kg/m^2^; * Microelement concentrations were measured in serum from 10-14 gestational week; ** Cases of pregnancy induced hypertension (PIH) and normotensive controls; *** *p*- value obtained using the Mann-Whitney U test (*p* < 0.05 was assumed to be significant).
