# Supplementary material for: First Trimester Serum Copper or Zinc Levels, and Risk of Pregnancy-Induced Hypertension
Source: Nutrients. 2019 Oct 16;11(10):2479. doi: 10.3390/nu11102479 (PMC6835641; doi:10.3390/nu11102479)
Supplement: Supplementary file 1 [file nutrients-11-02479-s001.zip › Table S3.docx]

**Table S3.** The odds ratios of pregnancy-induced hypertension for copper, zinc and Cu:Zn ratio levels in the whole cohort and subgroups, in univariate and multivariate logistic regression.

| **Risk of Pregnancy–Induced Hypertension (PIH)/Gestational Hypertension (GH)** | | | | | | | | | | | | | | | | | | | | | | | | | | | | |
| --- | --- | --- | --- | --- | --- | --- | --- | --- | --- | --- | --- | --- | --- | --- | --- | --- | --- | --- | --- | --- | --- | --- | --- | --- | --- | --- | --- | --- |
| **Quartile** | **Cases•** | | | | | **Controls** | | | | | | | **OR * 95%CI:); *p* **** | | |  | **AOR * (95%CI:); *p* **** | | | | | | | | | | |  |
| **Cu (µg/L) !** | | | | |  | |  | | | |  | | | |  | | |  | | | | | | | | | | |
| **Whole cohort- PIH cases (*N* = 484)** | | | | | | | |  | | | |  | | | |  | | |  | | | | |  | | | | |
| Q_1:_ 883.61–1540.58 | | | 33 | | | | 88 | | | | 1.52 (0.83–2.76); 0.174 | | | | |  | | **2.17 (1.14–4.16); 0.019** | | | | | | |  |  |  |  |
| Q_2:_ 1540.58–1733.78 | | | 37 | | | | 84 | | | | 1.78 (0.99–3.22); 0.056 | | | | |  | | **2.39 (1.28–4.49); 0.007** | | | | | | |  |  |  |  |
| Q_3:_ 1733.78–1937.46 | | | 27 | | | | 94 | | | | 1.16 (0.63–2.16); 0.637 | | | | |  | | 2.35 (0.71–2.57); 0.360 | | | | | | |  |  |  |  |
| Q_4:_ 1937.46–3956.76 | | | 24 | | | | 97 | | | | 1 | | | | |  | | 1 | | | | | | |  |  |  |  |
| **Subgroup of normal BMI (*N* = 265) #** | | | | | | | | | | | |  | | | |  | | | | |  | | |  | | | | |
| Q_1:_ 969.60–1486.63 | | | 17 | | | | 49 | | | | **2.97 (1.14–7.75); 0.026** | | | | |  | | **2.95 (1.05–8.27); 0.040** | | | | | | |  |  |  |  |
| Q_2:_ 1486.63–1667.50 | | | 18 | | | | 48 | | | | **3.21 (1.24–8.33); 0.016** | | | | |  | | **4.05 (1.44–11.43); 0.008** | | | | | | |  |  |  |  |
| Q_3:_ 1667.50–1838.35 | | | 12 | | | | 54 | | | | 1.91 (0.70–5.19); 0.208 | | | | |  | | 2.18 (0.75–6.39); 0.154 | | | | | | |  |  |  |  |
| Q_4:_ 1838.35–2500.75 | | | 7 | | | | 60 | | | | 1 | | | |  | | |  | | | | | | |  |  |  |  |
| **Whole cohort-only GH cases (*N* = 424)** | | | | | | | | | | | |  | | | |  | | |  | | | | |  | | | | |
| Q_1:_ 883.61–1541.95 | | | 30 | | | | 76 | | | | 1.60 (0.84–3.02); 0.150 | | | | | |  | **2.36 (1.19–4.68); 0.015** | | | | | | |  |  |  |  |
| Q_2:_ 1541.95–1735.86 | | | 32 | | | | 74 | | | | 1.75 (0.93–3.30); 0.083 | | | | | |  | **2.35 (1.20–4.60); 0.012** | | | | | | |  |  |  |  |
| Q_3:_ 1735.86–1937.46 | | | 23 | | | | 83 | | | | 1.12 (0.58–2.18); 0.735 | | | | | |  | 1.27 (0.64–2.52); 0.503 | | | | | | |  |  |  |  |
| Q_4:_ 1937.46–3956.76 | | | 21 | | | | 85 | | | | 1 | | | | | |  | 1 | | | | | | |  |  |  |  |
| **Zn (µg/L) ! ••** | | | | |  | | | |  | |  | | | | | |  |  | | | | | | | | | | |
| **Whole cohort– PIH cases (*N* = 484)** | | | | | | | | | |  | |  | | | |  | | | | | | | | | | | | |
| Q_1:_ 394.04 – 553.81 | | 30 | | | | | | 91 | | | | 1.21 (0.66–2.19); 0.542 | | | | |  | 1.17 (0.64–2.16); 0.612 | | | | | | | | |  |  |
| Q_2:_ 553.81 – 607.62 | | | 30 | | | | | 91 | | | | 1.21 (0.66–2.19); 0.542 | | | | |  | 1.18 (0.64–2.17); 0.605 | | | | | | | | |  |  |
| Q_3:_ 607.62 – 664.25 | | | 35 | | | | | 86 | | | | 1.49 (0.83–2.67); 0.184 | | | | |  | 1.48 (0.82–2.68); 0.199 | | | | | | | | |  |  |
| Q_4:_ 664.25 – 3238.90 | | | 26 | | | | | 95 | | | | 1 | | | | |  | 1 | | | | | | | | |  |  |
| **Subgroup of normal BMI (*N* = 265) #** | | | | | | | | | | | |  | | | |  | | | | | | | | | | |  |  |
| Q_1:_ 411.94 – 558.70 | | | | 12 | | 54 | | | | | | 1.13 (0.46–2.78); 0.788 | | | |  | | 0.92 (0.35–2.47); 0.873 | | | | | | | | |  |  |
| Q_2:_ 558.70 – 607.07 | | | | 13 | | 53 | | | | | | 1.25 (0.52–3.03); 0.623 | | | |  | | 1.04 (0.39–2.76); 0.937 | | | | | | | | |  |  |
| Q_3:_ 607.07 – 656.95 | | | | 18 | | 48 | | | | | | 1.91 (0.82–4.44); 0.133 | | | |  | | 1.58 (0.62–4.01); 0.338 | | | | | | | | |  |  |
| Q_4:_ 656.95 – 1069.54 | | | | 11 | | 56 | | | | | | 1 | | | |  | | 1 | | | | | | | | |  |  |
| **Cu:Zn ratios ! ••** | | | | |  | |  | | | | | | |  | | | | | |  | |  | | | |  |  |  |
| **Whole cohort-PIH cases (*N* = 484)** | | | | | | | | | |  | |  | | | |  | | | | | | |  |  | | |  |  |
| Q_1:_ 0.54 – 2.45 | | | 33 | | | | 88 | | | | | 1.44 (0.79–2.61); 0.230 | | | | |  | 1.61 (0.87–2.99); 0.129 | | | | | | | | |  |  |
| Q_2:_ 2.45 – 2.86 | | | 33 | | | | 88 | | | | | 1.44 (0.79–2.61); 0.230 | | | | |  | 1.61 (0.88–2.97); 0.126 | | | | | | | | |  |  |
| Q_3:_ 2.86 – 3.25 | | | 25 | | | | 96 | | | | | 1 | | | | |  | 1 | | | | | | | | |  |  |
| Q_4:_ 3.25 – 5.99 | | | 30 | | | | 91 | | | | | 1.27 (0.69–2.31); 0.444 | | | | |  | 1.15 (0.61–2.14); 0.669 | | | | | | | | |  |  |
| **Subgroup of normal BMI (*N* = 265) #** | | | | | | | | | | | |  | | | |  |  |  |  |  |  |  |  |  |  |  |  |  |
| Q_1:_ 1.18 – 2.36 | | | 20 | | | | 46 | | | | | **2.80 (1.17–6.73); 0.021** | | | | |  | **3.06 (1.17–8.03); 0.023** | | | | | | | | |  |  |
| Q_2:_ 2.36 – 2.76 | | | 13 | | | | 53 | | | | | 1.58 (0.63–4.00); 0.333 | | | | |  | 1.85 (0.67–5.08); 0.233 | | | | | | | | |  |  |
| Q_3:_ 2.76 – 3.12 | | | 12 | | | | 54 | | | | | 1.43 (0.56–3.67); 0.454 | | | | |  | 1.60 (0.58–4.46); 0.365 | | | | | | | | |  |  |
| Q_4:_ 3.12 – 4.65 | | | 9 | | | | 58 | | | | | 1 | | | | |  | 1 | | | | | | | | |  |  |

# normal pre-pregnancy BMI: body mass index 18.50- 24.99 kg/m2; ! Biomarker levels were measured in serum from the 10-14 gestational week and border values are included in the lower quartile; •Cases of PIH: pregnancy induced hypertension (GH and preeclampsia) or GH: gestational hypertension; **••**The results for GH cases were not statistically; * OR: crude odds ratio calculated in univariate logistic regression (in groups after matching several risk factors) and AOR: adjusted odds ratios calculated in multivariate logistic regression after adjusted for pre-pregnancy BMI and gestational age at recruitment (the BMI was excluded in the subgroup of normal BMI); CI: confidence intervals; ** p- value obtained using the Wald test (*p* < 0.05 was assumed to be significant).
